# Fluorocycline TP-271 Is Potent against Complicated Community-Acquired Bacterial Pneumonia Pathogens

**DOI:** 10.1128/mSphere.00004-17

**Published:** 2017-02-22

**Authors:** Trudy H. Grossman, Corey Fyfe, William O’Brien, Meredith Hackel, Mary Beth Minyard, Ken B. Waites, Jacques Dubois, Timothy M. Murphy, Andrew M. Slee, William J. Weiss, Joyce A. Sutcliffe

**Affiliations:** a Tetraphase Pharmaceuticals, Inc., Watertown, Massachusetts, USA; b International Health Management Associates, Inc., Schaumburg, Illinois, USA; c Southern Research Institute, Birmingham, Alabama, USA; d University of Alabama at Birmingham, Birmingham, Alabama, USA; e M360, Sherbrooke, Quebec, Canada; f Vivisource Laboratories, Waltham, Massachusetts, USA; g University of North Texas Health Science Center, Fort Worth, Texas, USA; JMI Laboratories

**Keywords:** TP-271, community-acquired bacterial pneumonia, fluorocycline

## Abstract

TP-271 is a novel, fully synthetic fluorocycline antibiotic in clinical development for the treatment of respiratory infections caused by susceptible and multidrug-resistant pathogens. TP-271 was active in MIC assays against key community respiratory Gram-positive and Gram-negative pathogens, including *Streptococcus pneumoniae* (MIC_90_ = 0.03 µg/ml), methicillin-sensitive *Staphylococcus aureus* (MSSA; MIC_90_ = 0.25 µg/ml), methicillin-resistant *S. aureus* (MRSA; MIC_90_ = 0.12 µg/ml), *Streptococcus pyogenes* (MIC_90_ = 0.03 µg/ml), *Haemophilus influenzae* (MIC_90_ = 0.12 µg/ml), and *Moraxella catarrhalis* (MIC_90_ ≤0.016 µg/ml). TP-271 showed activity (MIC_90_ = 0.12 µg/ml) against community-acquired MRSA expressing Panton-Valentine leukocidin (PVL). MIC_90_ values against *Mycoplasma pneumoniae*, *Legionella pneumophila*, and *Chlamydia pneumoniae* were 0.004, 1, and 4 µg/ml, respectively. TP-271 was efficacious in neutropenic and immunocompetent animal pneumonia models, generally showing, compared to the burden at the start of dosing, ~2 to 5 log_10_ CFU reductions against MRSA, *S. pneumoniae*, and *H. influenzae* infections when given intravenously (i.v.) and ~1 to 4 log_10_ CFU reductions when given orally (p.o.). TP-271 was potent against key community-acquired bacterial pneumonia (CABP) pathogens and was minimally affected, or unaffected, by tetracycline-specific resistance mechanisms and fluoroquinolone or macrolide drug resistance phenotypes.

**IMPORTANCE** Rising resistance rates for macrolides, fluoroquinolones, and β-lactams in the most common pathogens associated with community-acquired bacterial pneumonia (CABP) are of concern, especially for cases of moderate to severe infections in vulnerable populations such as the very young and the elderly. New antibiotics that are active against multidrug-resistant *Streptococcus pneumoniae* and *Staphylococcus aureus* are needed for use in the empirical treatment of the most severe forms of this disease. TP-271 is a promising new fluorocycline antibiotic demonstrating *in vitro* potency and nonclinical efficacy by intravenous and oral administration against the major pathogens associated with moderate to severe CABP.

## Abstract (precis)

Rising resistance rates for macrolides, fluoroquinolones, and β-lactams in the most common pathogens associated with community-acquired bacterial pneumonia (CABP) are of concern, especially for cases of moderate to severe infections in vulnerable populations such as the very young and the elderly. New antibiotics that are active against multidrug-resistant *Streptococcus pneumoniae* and *Staphylococcus aureus* are needed for use in the empirical treatment of the most severe forms of this disease. TP-271 is a promising new fluorocycline antibiotic demonstrating *in vitro* potency and nonclinical efficacy by intravenous and oral administration against the major pathogens associated with moderate to severe CABP.

## INTRODUCTION

Community-acquired bacterial pneumonia (CABP) is a serious condition associated with mortality rates estimated to be as high as 12 to 14% for hospitalized individuals and 25 to 40% for those admitted to intensive care units (1–3). Lower respiratory tract infections were the second greatest cause of deaths and years of life lost in 2013 as reported by The Global Burden of Disease Study, with the highest incidence occurring in children <5 years and adults >65 years (1, 4). The Centers for Disease Control estimated that 30% of severe *Streptococcus pneumoniae* infections are fully resistant to one or more antibiotics; drug-resistant *S. pneumoniae* infections complicate treatment and cause approximately 7,000 deaths per year (5). Excess medical costs associated with treating drug-resistant *S. pneumoniae* infections were estimated at approximately $96 million per year.

The bacterial etiology of CABP varies with severity of disease; however, *S. pneumoniae* is the most frequent cause of CABP across all levels of severity (6). The incidence of infections by *Staphylococcus aureus* and *Legionella* spp. increases with more-severe CABP, whereas *Haemophilus influenzae*, *Mycoplasma pneumoniae*, and *Chlamydia pneumoniae* are generally associated with mild to moderate CABP (6, 7). Recently, large medical centers in the United States saw a dramatic increase in the incidence of infections caused by *S. aureus*, including those caused by methicillin-resistant *S. aureus* (MRSA) strains, once considered rare in CABP (8, 9). A survey of 59 United States hospitals, involving 4,543 patients with culture-positive pneumonias between January 2002 and January 2004, identified *S. aureus* and MRSA as potential pathogens in 25.5% and 8.9% of the cases, respectively (8). Further, in this study, *S. aureus* was identified by logistic regression analysis as the only pathogen independently associated with mortality. Most of the MRSA strains causing health care-associated pneumonia, hospital-acquired bacterial pneumonia, and ventilator-associated bacterial pneumonia are hospital-acquired MRSA (HA-MRSA) strains containing staphylococcal cassette chromosome *mec* type I (SCC*mec* I) to SCC*mec* III (10). A new variant of MRSA, identified as community-acquired MRSA (CA-MRSA) containing SCC*mec* IV, has emerged globally as a potent pulmonary pathogen (10). This strain type typically carries a bacteriophage encoding Panton-Valentine leukocidin (PVL), a toxin that destroys polymorphonuclear leukocytes and is associated with tissue necrosis and increased virulence (11, 12). The increased incidence of CA-MRSA infections in more-severe cases of CABP limits the empirical use of fluoroquinolones, macrolides, and most currently marketed β-lactams due to high resistance rates (13, 14).

Although there are numerous therapies for *S. pneumoniae* infection, including oral drugs in the macrolide, fluoroquinolone, and β-lactam classes, resistance to β-lactams and macrolides is increasing (6). Resistance to currently approved antibiotics in other respiratory pathogens is also increasing. Global surveillance by the Tigecycline Evaluation and Surveillance Trial (TEST) in 2004 to 2013 found that penicillin-resistant *S. pneumoniae* (PRSP) comprised 14.8% of the *S. pneumoniae* isolates and that the percentages were highest in the Middle East (24.7%), Africa (28.1%), and the Asia-Pacific Rim (30.1%) (15). Erythromycin resistance during the same period was 32.7% and was highly correlated with penicillin resistance. TEST also found that the global rate of β-lactamase production among *H. influenzae* isolates collected from 2004 to 2013 was 20.1%. In the AWARE ceftaroline surveillance program, 96.4% of *Moraxella catarrhalis* isolates collected in the United States from 2008 to 2010 were β-lactamase producers (16). The increasing prevalence of antimicrobial resistance in *S. pneumoniae*, *H. influenzae*, and *M. catarrhalis* significantly impacts the utility of currently available antibiotics (17). Macrolide resistance in *M. pneumoniae* has increased worldwide, with >90% of clinical isolates from Japan and China and ~20% of isolates in some European countries having high-level resistance to azithromycin (18). A surveillance study performed in the United States from 2012 to 2014 reported a macrolide resistance rate of 13.2% (19). Macrolide resistance in *M. pneumoniae* is clinically significant, often requiring switching to other drug classes such as tetracyclines or fluoroquinolones (18).

**FIG 1:**
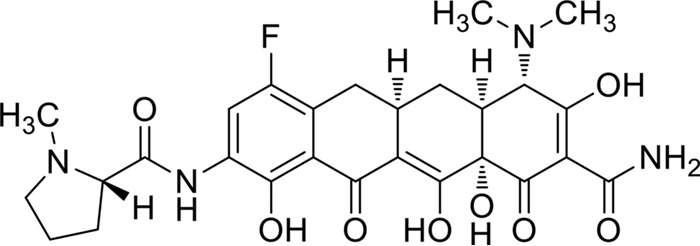
Chemical structure of TP-271.

Tetracycline antibiotics are well known for their broad spectrum, which includes a wide range of Gram-positive and Gram-negative bacteria, spirochetes, and obligate intracellular bacteria. TP-271, a novel fluorocycline antibiotic of the tetracycline class (Fig. 1), is a candidate for the treatment of serious infections, including those caused by multidrug-resistant pathogens. On the basis of *in vitro* and *in vivo* evaluations, TP-271 shows potential to treat key susceptible and resistant organisms associated with moderate to severe CABP.

## RESULTS

### TP-271 mechanism of action.

The site of action of tetracycline drugs such as TP-271 is the 30S ribosomal subunit; drug binding interferes with access of aminoacyl-tRNA to the A-site on the mRNA-ribosome complex, preventing new amino acid addition and peptide chain growth (20, 21). TP-271 showed potent mechanism-based antitranslational activity in an *in vitro* coupled transcription/translation (TnT) assay in the presence and absence of the tetracycline-specific ribosomal protection protein Tet(M), which confers high-level resistance to tetracycline in bacteria (22). The half-maximal inhibitory concentration (IC_50_) determined in the TnT assay for TP-271 was 0.18 ± 0.08 µg/ml; in comparison, the IC_50_ for tetracycline was 1.1 ± 0.07 µg/ml and for the nontetracycline translation inhibitor linezolid the IC_50_ was 1.3 ± 0.28 µg/ml. In the presence of Tet(M), the IC_50_ for TP-271 was unaffected (0.13 ± 0.04 µg/ml) whereas the IC_50_ for tetracycline increased by ~5-fold (5.8 ± 1.1 µg/ml), consistent with the tetracycline resistance seen with Tet(M)-expressing organisms. Thus, TP-271 is distinguished by its being a Tet(M)-insensitive novel tetracycline.

### Activity of TP-271 against CABP pathogens *in vitro*.

TP-271 was active against key community respiratory Gram-positive pathogens (Table 1), including *S. pneumoniae* (MIC_90_ = 0.03 µg/ml), methicillin-sensitive *S. aureus* (MSSA; MIC_90_ = 0.25 µg/ml), MRSA (MIC_90_ = 0.12 µg/ml), and *Streptococcus pyogenes* (MIC_90_ = 0.03 µg/ml). TP-271 was active (MIC_90_ = 0.12 µg/ml) against CA-MRSA expressing PVL. As shown by the MIC_90_ data, TP-271 was ≥1,000-fold more potent than tetracycline against *S. pneumoniae* and *S. pyogenes* and 128-fold more potent against *S. aureus*. TP-271 was also potent against respiratory Gram-negative pathogens *H. influenzae* (MIC_90_ = 0.12 µg/ml) and *M. catarrhalis* (MIC_90_ = ≤0.016 µg/ml).

**TABLE 1:** Susceptibilities of CABP pathogens to TP-271 and comparators^a^

Organism	No. of isolates	MIC_50_/MIC_90_ (μg/ml), MIC range (μg/ml)						
		TP-271	TET	TGC	MAC^g^	FQ^h^	LZD	VAN
*Streptococcus* *pneumoniae*	267	≤0.016/0.03, ≤0.016–0.03	32/>32,^b^ ≤0.016–>32	≤0.016/≤0.016,^c^ ≤0.016–≤0.016	>32/>32, ≤0.016–>32	1/1, 0.25–32	1/1,^b^ 0.12–2	0.5/0.5,^b^ ≤0.016–0.5
*Streptococcus* *pyogenes*	100	≤0.016/0.03, ≤0.016–0.03	0.5/>32, 0.12–32	≤0.016/≤0.016,^d^ ≤0.016–0.06	0.06/>32, ≤0.016–>32	0.5/1, 0.25–2	1/2, 0.5–2	0.5/0.5, 0.25–0.5
*Staphylococcus* *aureus*	155	0.06/0.25, ≤0.03–1	≤2/32, 0.06–32	0.12/0.25, ≤0.016–0.5	>4/>4, 0.25–4	>4/>4, ≤0.12–>4	2/4, 0.5–64	1/1, ≤0.5–8
MRSA	124	0.06/0.12, ≤0.016–1	≤2/32, 0.06–32	0.12/0.25, ≤0.016–0.5	>4/>4, 0.25–4	>4/>4, ≤0.12–>4	2/4, 1–64	1/1, ≤0.5–8
MRSA, PVL^+^	25	0.06/0.12, 0.06–0.12	≤2/≤2, ≤2–16	0.12/0.12, 0.06–0.25	>4/>4, 1–>4	2/>4, ≤0.12–>4	2/2, 1–4	1/1, ≤0.5–1
MSSA	31	0.12/0.25, ≤0.03–0.25	≤2/≤2, ≤2–32	0.12/0.25, 0.03–0.25	1/>4, 0.5–4	0.25/0.5, ≤0.12–>4	2/4, 0.5–4	1/1, ≤0.5–1
*Haemophilus* *influenzae*	65	0.03/0.12, ≤0.016–0.25	0.5/4, 0.12–16	0.06/0.25, ≤0.016–0.5	8/8, 0.06–16	≤0.016/0.03, ≤0.016–0.12	8/16, 4–32	>32/>32,^e^ 16–>32
*Moraxella* *catarrhalis*	57	≤0.016/≤0.016, ≤0.016–0.031	0.5/32, 0.12–32	≤0.016/0.031, ≤0.016–0.12	0.06/0.25, ≤0.016–4	0.03/0.06, 0.03–0.12	8/8, 2–32	>32/>32,^f^ 16–>32

a TET, tetracycline; TGC, tigecycline; MAC, macrolide (erythromycin, azithromycin, or clarithromycin); FQ, fluoroquinolone (ciprofloxacin or levofloxacin); LZD, linezolid; VAN, vancomycin.

b 256 *S. pneumoniae* isolates.

c 137 *S. pneumoniae* isolates.

d 64 *S. pyogenes* isolates.

e 51 *H. influenzae* isolates.

f 43 *M. catarrhalis* isolates.

g Isolates were tested with either azithromycin, erythromycin, or clarithromycin, and all MIC values were pooled for MAC MIC_50_ and MIC_90_ determinations.

h isolates were tested with either ciprofloxacin or levofloxacin, and all MIC values were pooled for FQ MIC_50_ and MIC_90_ determinations.

For hospitalized CABP patients on the general wards, the Infectious Diseases Society of America/American Thoracic Society guidelines currently recommend a respiratory fluoroquinolone or the combination of a β-lactam and a macrolide (7). For patients with severe CABP requiring intensive care unit admission, the guidelines recommend a β-lactam plus either azithromycin or a respiratory fluoroquinolone; if MRSA is a concern, either vancomycin or linezolid should be added (7). TP-271 retained good antibacterial potency against subsets of *S. pneumoniae* resistant to penicillin (MIC_90_ = 0.03 μg/ml) and macrolides (MIC_90_ = 0.03 μg/ml) and against MRSA displaying resistance to fluoroquinolones (MIC_90_ = 0.25 μg/ml), macrolides (MIC_90_ = 0.25 μg/ml), and linezolid (MIC range, ≤0.016 to 0.5 μg/ml) (Table 2). TP-271 was also active against MRSA isolates displaying nonsusceptibility to daptomycin (MIC range, ≤0.016 to 0.25 μg/ml), a drug used in the treatment of serious Gram-positive infections, excluding pneumonia (Table 2).

**TABLE 2:** Susceptibilities of drug-resistant MRSA and *S. pneumoniae* to TP-271 and comparators^a^

Organism	Phenotype^b^	MIC_50_/MIC_90_ or range (µg/ml)							
		TP-271	LZD	DAP	VAN	TGC	TET	MAC^h^	FQ^i^
*S. pneumoniae*	Pen-R^c^ (*n* = 125)	≤0.016/0.03	1/1	ND	0.5/0.5	≤0.016/≤0.016^d^	32/>32	>32/>32	1/1
	MAC-R (*n* = 209)	≤0.016/0.03	1/1^g^	ND	0.5/0.5^*g*^	≤0.016/≤0.016^e^	32/>32^f^	>32/>32	1/1^*g*^
									
MRSA	FQ-R (*n* = 97)	0.06/0.25	2/4	0.25/2	1/1	0.12/0.5	1/32	>32/>32	8/>32
	MAC-R (*n* = 101)	0.06/0.25	2/4	0.25/1	1/1	0.12/0.5	1/>32	>32/>32	8/>32
	LZD-R (*n* = 9)	≤0.016–0.5	8–64	0.12–0.5	0.5–2	0.031–0.5	0.5–>32	0.5–>32	8–>32
	Dap-NS (*n* = 5)	≤0.016–0.25	2–2	2–4	2–8	≤0.016–0.5	0.063–>32	>32–>32	0.5–16

a TET, tetracycline; TGC, tigecycline; MAC, macrolide (erythromycin, azithromycin, or clarithromycin); FQ, fluoroquinolone (ciprofloxacin or levofloxacin); LZD, linezolid; VAN, vancomycin.

b MAC-R, resistant to one or more of azithromycin, erythromycin, and clarithromycin; FQ-R, resistant to levofloxacin or ciprofloxacin or both; LZD-R, resistant to linezolid; DAP-NS, nonsusceptible to daptomycin. Resistance (R) and nonsusceptibility (NS) were determined as defined by CLSI.

c Penicillin MIC, ≥2 µg/ml.

d 58 *S. pneumoniae* isolates.

e 82 *S. pneumoniae* isolates.

f 201 *S. pneumoniae* isolates.

g 185 *S. pneumoniae* isolates.

h Isolates were tested with either azithromycin, erythromycin, or clarithromycin, and all MIC values were pooled for MIC_50_ and MIC_90_ determinations.

i Isolates were tested with either ciprofloxacin or levofloxacin, and all MIC values were pooled for MIC_50_ and MIC_90_ determinations.

As recommended in the August 2016 FDA publication of guidance for microbiological data for systemic antibacterial drug products (23), TP-271 was tested for retention of microbiological activity in the presence of 5% bovine pulmonary surfactant and found to be equally active against *S. aureus* ATCC 29213 (MIC = 0.063 µg/ml in cation-adjusted Mueller-Hinton broth [ca-MHB]; MIC = 0.031 µg/ml in ca-MHB + 5% bovine pulmonary surfactant). The MIC of the positive control, daptomycin, was elevated by 512-fold in ca-MHB + 5% bovine pulmonary surfactant, as expected (24).

### Activity of TP-271 against atypical respiratory pathogens.

TP-271 was tested against atypical pathogens commonly associated with CABP: *L. pneumophila*, *M. pneumoniae*, and the obligate intracellular pathogen *C. pneumoniae* (Table 3). TP-271 showed an MIC_90_ of 1 µg/ml against a panel comprised of 20 isolates of *L. pneumophila* serogroup 1 and 10 isolates each of serogroups 2 to 6; TP-271 was found to be similarly potent against all serogroups when assessed individually (25). When tested by agar dilution in buffer yeast extract (BYE), it was noted that the TP-271 and tetracycline MIC values for the quality control strain *S. aureus* ATCC 29213 were 128-fold and 64-fold higher, respectively, compared to results in cation-adjusted Mueller-Hinton agar after 48 h of incubation, the duration of incubation for *L. pneumophila* testing (data not shown). This finding suggested that the BYE medium used in the testing of *L. pneumophila* may have reduced the activity of TP-271 and inflated the MIC values for *L. pneumophila*.

**TABLE 3:** Susceptibilities of atypical pathogens to TP-271 and comparators

Organism	No. of isolates	MIC_50_/MIC_90_ (μg/ml), MIC range (μg/ml)				
		TP-271	TET^a^	TGC	MAC^b^	LVX
*Legionella pneumophila*	70	0.25/1, ≤0.004–2	4/8, 0.5-8	ND^c^	0.25/0.5, 0.06–1	ND
*Chlamydia pneumoniae*	10	4/4, 2–4	0.25/0.25, 0.12–0.25	ND	0.25/0.25, 0.12–0.25	ND
*Mycoplasma pneumoniae*	20	0.001/0.004, 0.0005–0.008	0.063/0.12, 0.032–0.12	0.031/0.031, 0.016–0.12	0.000063/8, 0.000032–16	0.5/0.5, 0.25–0.5

a Doxycycline for *C. pneumoniae* and *M. pneumoniae*, tetracycline for *L. pneumophila*.

b Azithromycin for *C. pneumoniae* and *M. pneumoniae*, erythromycin for *L. pneumophila*.

c ND, not determined.

The TP-271 MIC_90_ was 0.004 μg/ml (range, 0.0005 to 0.008 μg/ml) against a panel of 20 clinical isolates of *M. pneumoniae* which included 4 organisms that were macrolide resistant (azithromycin MICs, ≥4 μg/ml) (Table 3).

TP-271 was tested against 10 human isolates of* C. pneumoniae* in an intracellular infection assay with HEp-2 cells and showed an MIC_90_ of 4 µg/ml (Table 3). Similar to the interference from media observed in *L. pneumophila* assays, the MIC values of TP-271 were 4- to 32-fold higher in Eagle’s minimum essential medium (EMEM) than in ca-MHB for the quality control organisms *S. aureus* ATCC 29213, *Escherichia coli* ATCC 25922, *Klebsiella pneumoniae* ATCC 13883, and *Pseudomonas aeruginosa* ATCC 27853 tested by broth microdilution assay (data not shown). These results suggested that the use of EMEM reduced the activity of TP-271 and artificially raised MIC values.

### The effect of common tetracycline resistance determinants on *in vitro* activity of TP-271.

Given the widespread dissemination of tetracycline resistance determinants in clinical isolates (26), it is important that a new tetracycline-class antibiotic be active against the major tetracycline resistance mechanisms. TP-271 was tested against a previously described panel of isogenic strains overexpressing major tetracycline resistance genes from an arabinose-inducible promoter on a plasmid in laboratory *E. coli* strain DH10B (22). The overexpressed genes encoded two major efflux mechanisms found in Gram-negative bacteria, Tet(A) and Tet(B); a major Gram-positive efflux mechanism, Tet(K); a major ribosomal protection mechanism, Tet(M); and Tet(X), a secreted flavin-dependent monooxygenase, originally identified in *Bacteroides fragilis*, capable of covalently inactivating tetracycline (22, 27, 28). Compared to the negative-control strain expressing *lacZ*, overexpression of all *tet* resistance genes had a pronounced effect on tetracycline and minocycline susceptibility, increasing MIC values by ≥32-fold and by 2-fold to >64-fold, respectively (Table 4). Overexpression of *tet*(K), *tet*(B), and *tet*(M) had no significant effect on the TP-271 MIC (values were within 2-fold of the control value); however, a 16-fold to 32-fold increase was observed in the presence of overexpression of *tet*(A) and *tet*(X). The highest MIC observed for TP-271 in the presence of an overexpressed *tet* gene was 4 µg/ml. As expected, the activity of the nontetracycline control, ceftriaxone, was not impacted by *tet* gene expression.

**TABLE 4:** Activity of TP-271 against *E. coli* expressing recombinant tetracycline resistance genes

Strain	*tet* or *lac*Z gene expressed	MIC (µg/ml)^a^			
		TP-271	MIN	TET	CRO
EC969	*tet*(M)	0.12	>32	128	0.12
EC970	*tet*(K)	0.25	1	128	0.06
EC1082	*tet*(A)	2	8	>128	0.12
EC1083	*tet*(B)	0.25	8	>128	0.06
EC1153	*tet*(X)	4	2	128	0.12
EC971	*lac*Z	0.12	0.5	4	0.12

a MIN, minocycline; TET, tetracycline; CRO, ceftriaxone.

### The effect of upregulated chromosomally encoded efflux pumps in *S. aureus* on TP-271 activity.

TP-271 was evaluated against previously described *S. aureus* clinical isolates with upregulated expression of *norA* (29, 30) and *mepA* (31, 32), genes encoding intrinsic efflux pumps conferring resistance to quinolones (NorA and MepA) and tigecycline (MepA). The MIC value of TP-271 for both *norA*-overexpressing mutant SA982 and the corresponding SA981 parental strain was 0.06 µg/ml; however, an 8-fold increase in the MIC was noted for TP-271 in *mepA*-overexpressing mutant SA984 (MIC = 0.12 µg/ml) versus the corresponding parental SA983 strain (MIC = 0.016 µg/ml) (Table 5). The MIC value for the tigecycline comparator was 4-fold higher in the *norA* mutant and 16-fold higher in the *mepA* mutant than in the corresponding parental strain.

**TABLE 5:** TP-271 activity against *S. aureus* in the presence of overexpressed NorA or MepA efflux pumps

Compound	MIC (µg/ml)^a^			
	SA981 (wild type)	SA982 (*norA*^*++*^)	SA983 (wild type)	SA984 (*mepA++*)
TP-271	0.06	0.06	0.016	0.12
Tigecycline	0.06	0.25	0.06	1
Ciprofloxacin	0.5	32	2	4

a Double plus signs (^*++*^) indicate overexpression of gene.

### Activity of TP-271 in murine respiratory infection models.

TP-271 administered either intravenously (i.v.) or orally (p.o.) reduced the bacterial burden in the lung, versus the control group results at the start of dosing, in murine pneumonia models with MRSA, *S. pneumoniae*, and *H. influenzae*.

### Neutropenic mouse MRSA pneumonia model.

**FIG 2:**
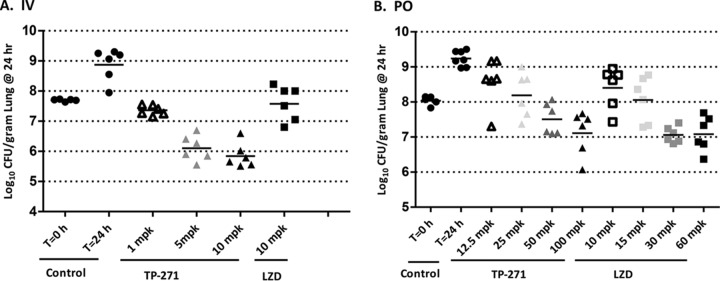
Activity of TP-271 administered i.v. and p.o. in a neutropenic murine MRSA *tet*(M) pneumonia model. Each symbol represents an individual mouse, and horizontal lines indicate the means. (A) TP-271 IV (i.v. administration). Untreated controls at 0 and 24 h, relative to the start of treatment, closed circles; TP-271, 1 mg/kg (1 mpk) (open triangles), 5 mg/kg (gray triangles), and 10 mg/kg (closed triangles); linezolid, 10 mg/kg (closed squares). (B) TP-271 PO (p.o. administration). Untreated controls at 0 and 24 h, relative to the start of treatment, closed circles; TP-271, 12.5 mg/kg (open triangles), 25 mg/kg (light gray triangles), 50 mg/kg (dark gray triangles), and 100 mg/kg (closed triangles); linezolid, 10 mg/kg (open squares), 15 mg/kg (light gray squares), 30 mg/kg (dark gray squares), and 60 mg/kg (closed squares).

In a neutropenic lung infection model challenged with tetracycline-resistant *tet*(M) MRSA strain SA191, TP-271 dosed i.v. at 2 and 12 h postinfection at 1, 5, and 10 mg/kg of body weight produced mean log_10_ CFU reductions of 0.62 ± 0.55, 1.58 ± 0.42, and 1.84 ± 0.41, respectively, exceeding the response seen with linezolid administered at 10 mg/kg, which was static (0.10 ± 0.58 log_10_ CFU reduction; Fig. 2). TP-271 administered p.o. at 25 mg/kg gave static results (0.17 ± 0.64 log_10_ CFU increase), and 50 and 100 mg/kg produced mean log_10_ CFU reductions of 0.52 ± 0.44 and 0.91 ± 0.63, respectively. This compared favorably to the response seen with linezolid administered p.o. at 15 mg/kg, which was static (0.04 ± 0.65 log_10_ CFU increase), and with 30 and 60 mg/kg, which produced mean log_10_ CFU reductions of 0.97 ± 0.23 and 0.94 ± 0.5, respectively.

### Neutropenic mouse *S. pneumoniae* pneumonia model.

**FIG 3:**
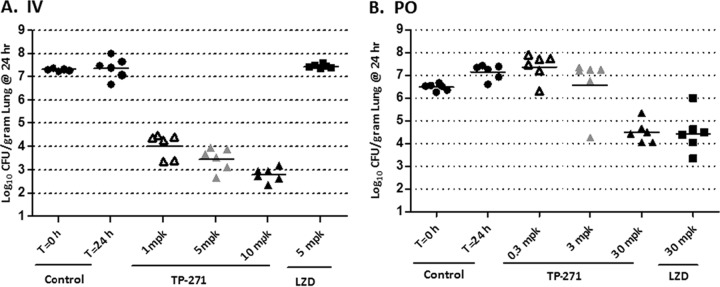
Activity of TP-271 administered i.v. and p.o. in a neutropenic murine *S. pneumoniae tet*(M) pneumonia model. Each symbol represents an individual mouse, and horizontal lines indicate the means. (A) TP-271 IV (i.v. administration). Untreated controls at 0 and 24 h, relative to the start of treatment, closed circles; TP-271, 1 mg/kg (open triangles), 5 mg/kg (gray triangles), and 10 mg/kg (closed triangles); linezolid, 5 mg/kg (closed squares). (B) TP-271 PO (p.o. administration). Untreated controls at 0 and 24 h, relative to the start of treatment, closed circles; TP-271, 0.3 mg/kg (open triangles), 3 mg/kg (gray triangles), and 30 mg/kg (closed triangles); linezolid, 30 mg/kg (closed squares).

In a neutropenic lung infection model challenged with tetracycline-resistant *tet*(M) *S. pneumoniae* strain SP160, TP-271 dosed 2 and 12 h postinfection at 1, 5, and 10 mg/kg i.v. produced mean log_10_ CFU reductions of 3.26 ± 0.53, 3.83 ± 0.50, and 4.51 ± 0.29, respectively (Fig. 3). In contrast, the effect seen with linezolid administered i.v. at 5 mg/kg was static (0.13 ± 0.08 mean log_10_ CFU increase). TP-271 administered p.o. at 0.3 mg/kg produced a 0.90 ± 0.58 mean log_10_ CFU increase; 3 mg/kg produced a static effect (0.68 ± 0.24 mean log_10_ CFU reduction). The 30 mg/kg dose produced a mean log_10_ CFU reduction of 1.97 ± 0.48, which was comparable to results seen with linezolid administered p.o. at 30 mg/kg, which produced a mean log_10_ CFU reduction of 1.99 ± 0.87.

### Immunocompetent mouse *S. pneumoniae* pneumonia model.

**FIG 4:**
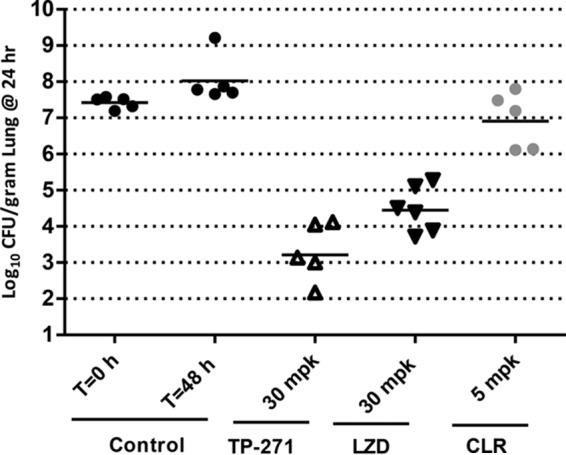
Activity of TP-271 administered p.o. in an immunocompetent murine *S. pneumoniae* pneumonia model. Each symbol represents an individual mouse, and horizontal lines indicate the means. Untreated controls at 0 and 48 h, relative to the start of treatment, closed circles; TP-271, 30 mg/kg (open triangles); linezolid, 30 mg/kg (inverted closed triangles); clarithromycin, 5 mg/kg (gray circles).

In immunocompetent mice challenged intranasally with macrolide-susceptible *S. pneumoniae* strain SP514, TP-271 dosed 5, 24, and 36 h postinfection with 30 mg/kg TP-271 p.o. produced a 4.13 ± 0.81 mean log_10_ reduction in CFU, exceeding the 2.95 ± 0.63 mean log_10_ reduction produced by linezolid given p.o. at the same dose (Fig. 4). In this model, the effect of administration of 5 mg/kg of clarithromycin p.o. was static (0.48 ± 0.78 mean log_10_ CFU reduction).

### Immunocompetent rat *H. influenzae* pneumonia model.

**FIG 5:**
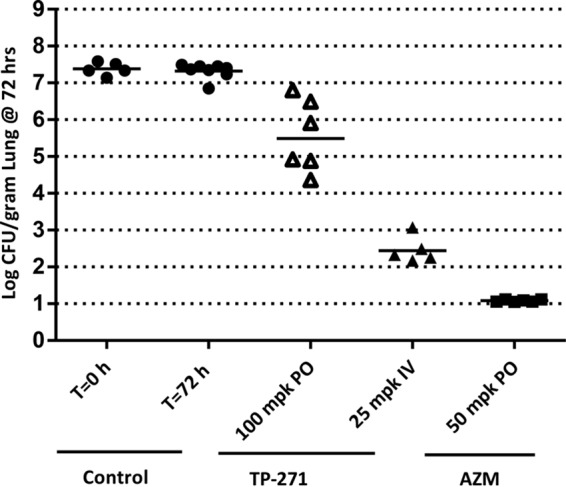
Activity of TP-271 administered i.v. and p.o. in an immunocompetent rat *H. influenzae* pneumonia model. Each symbol represents an individual mouse, and horizontal lines indicate the means. Untreated controls at 0 and 72 h, relative to the start of treatment, closed circles; TP-271 p.o., 100 mg/kg (open triangles), TP-271 i.v., 25 mg/kg (closed triangles); azithromycin, 50 mg/kg (closed squares).

In a rat lung infection model challenged with *H. influenzae* HI551, TP-271 dosed 100 mg/kg p.o. or 25 mg/kg i.v. at 5, 24, and 48 h postinfection produced 1.82 ± 0.98 and 4.92 ± 0.36 mean log_10_ reductions in CFU, respectively (Fig. 5). Azithromycin at 50 mg/kg p.o. produced a 6.30 ± 0.03 log_10_ CFU reduction.

## DISCUSSION

Recent FDA guidelines (23) indicate that new antibacterial drugs for CABP must have nonclinical data showing activity against the most commonly implicated pathogens, i.e., *S. pneumoniae*, *H. influenzae*, *S. aureus*, and *M. catarrhalis*, and for moderate to serious CABP, coverage of *L. pneumophila* is critical for empirical use. TP-271 meets these criteria, demonstrating good *in vitro* and *in vivo* potency against key susceptible and drug-resistant causative pathogens for this indication. The activity of TP-271 was minimally affected, or unaffected, by tetracycline-specific, fluoroquinolone, or macrolide resistances. MIC_90_ values for TP-271 were 0.03 µg/ml for all streptococci, the most common CABP pathogens, regardless of resistance phenotype. TP-271 was also active against MSSA and MRSA (MIC_90_ = 0.12 to 0.25 µg/ml), including community-acquired MRSA expressing PVL toxin. Against *H. influenzae* and *M. catarrhalis*¸TP-271 MIC_90_ values were 0.12 and ≤0.016 µg/ml, respectively. Good activity was also demonstrated against *M. pneumoniae*, with all MICs being ≤0.008 μg/ml, including those against macrolide-resistant organisms. TP-271 was active against *C. pneumoniae* (MIC_90_ = 4 µg/ml) and *L. pneumophila* (MIC_90_ = 1 µg/ml) despite potential interference from assay conditions. TP-271 was efficacious when administered i.v. and p.o. versus MRSA, *S. pneumoniae*, and *H. influenzae* in rodent pneumonia models, demonstrating potential as both an i.v. treatment and an oral treatment for CABP. The spectrum of activity of TP-271, along with the results from the animal infection models of pneumonia, makes it a promising candidate for development as an antibiotic for treatment of moderate to severe CABP.

## MATERIALS AND METHODS

### Antibiotics.

Commercial-grade antibiotics were obtained from the USP (Rockville, MD), ChemPacific Corp. (Baltimore, MD), or Sigma-Aldrich (St. Louis, MO). TP-271 was synthesized at Tetraphase Pharmaceuticals as described previously (33).

### *In vitro Escherichia coli* coupled transcription and translation (TnT) assay.

TP-271 and comparators were assayed for bacterial translation inhibition with a firefly luciferase readout, as described previously (22).

### Susceptibility testing of aerobic pathogens.

All MIC assays were performed by broth microdilution at Tetraphase Pharmaceuticals, International Health Management Associates (IHMA), and Mount Sinai Hospital (Toronto, Ontario, Canada) per Clinical and Laboratory Standards Institute (CLSI) guidelines (34). Isolates were recent and demographically diverse and were obtained from Eurofins Medinet (Chantilly, VA), IHMA, or the Canadian Bacterial Surveillance Network collection at Mount Sinai Hospital. For testing in the presence of pulmonary surfactant, MIC assays were performed with the addition of 5% beractant (Survanta; AbbVie, Inc., North Chicago, IL) and incubated at 37°C with agitation. Antibiotic resistance was determined per CLSI breakpoints (35).

### Susceptibility testing of atypical pathogens.

The *L. pneumophila* human respiratory isolates used were collected from 1992 to 2010 and were identified by standard methods as previously described (36). Seventy *L. pneumophila* isolates from serogroups 1 to 6 were tested by CLSI agar dilution methodology (34) at M360 (Sherbrooke, Quebec, Canada) using BYE with original *Legionella* growth supplement (Oxoid Canada, Nepean, Ontario, Canada) (per 100 ml of medium: buffer/potassium hydroxide [1.0 g], ferric pyrophosphate [0.025 g], l-cysteine HCl [0.04 g], and α-ketoglutarate [0.1 g]).

Low-passage-number clinical isolates of *M. pneumoniae* isolated between 1999 and 2012 were tested by broth microdilution at the University of Alabama, Birmingham (UAB), Diagnostic Mycoplasma Laboratory using SP4 broth per CLSI methodology (37).

The 10 *C. pneumoniae* human isolates used in this study were obtained from the University of Washington (Seattle, WA), UAB, and the American Type Culture Collection (ATCC; Manassas, VA). Each *C. pneumoniae* isolate was propagated and screened for susceptibility at the Southern Research Institute (Birmingham, AL) using HEp-2 cells (ATCC) (38). HEp-2 cells were grown in Eagle’s minimum essential medium (EMEM; ATCC, Manassas, VA) supplemented with 10% fetal bovine serum (FBS; Atlanta Biologicals, Flowery Branch, GA) on poly-l-lysine coated coverslips in 12-well tissue culture-treated plates. A HEp-2 cell suspension (1 ml) was pipetted at ~2.0 × 10^5^ cells/ml onto each coverslip and incubated at 35°C in 5% CO_2_ for 24 h to allow adherence. After incubation, the culture medium was removed and cells were rinsed with 1 ml HEPES-buffered sterile saline solution. Approximately 1.0 × 10^6^ *C. pneumoniae* elementary bodies in sucrose/phosphate/glutamate buffer (Sigma-Aldrich, St. Louis, MO) were added per well for a multiplicity of infection of 10. Plates were centrifuged at 900 × *g* for 60 min at ambient temperature, and supernatants were aspirated from each well and replaced with 1 ml of EMEM containing 1 μg/ml cycloheximide with or without a test compound dilution. After incubation at 35°C in CO_2_ for 72 h, the medium was removed and 1 ml of 95% ethanol was added. After 10 min, coverslips were placed on a microscope slide and inclusion bodies were detected using fluorescein-conjugated anti-*Chlamydia* monoclonal antibody from a PathoDx *Chlamydia* confirmation kit (ThermoScientific, Waltham, MA). The MIC was defined as the lowest concentration of test compound resulting in a >50% reduction in the number of inclusions after 3 days of treatment. Controls were (i) noninfected cells, (ii) infected cells without drug, and (iii) infected cells treated with doxycycline and azithromycin.

### Susceptibility testing against *E. coli* DH10B overexpressing recombinant tetracycline resistance genes.

Evaluation of TP-271 and comparators against laboratory *E. coli* strain DH10B overexpressing tetracycline resistance genes was performed as previously described (22).

### Susceptibility testing against *S. aureus* strains overexpressing *norA* and *mepA* efflux pump genes.

Evaluation of TP-271 and comparators against *S. aureus* overexpressing chromosomally encoded multidrug resistance efflux pumps was performed as previously described (39).

### Animal infection models.

All studies were performed at Vivisource Laboratories, Waltham, MA, with the exception of the neutropenic *S. pneumoniae* model using p.o. administration, which was performed at the University of North Texas Health Science Center. All procedures followed Institutional Animal Care and Use Committee-approved protocols. Pathogen-free female mice, CD-1 or BALB/c, and male Sprague-Dawley rats were purchased from Charles River Laboratories, Inc. (Wilmington, MA), and were acclimated for a minimum of 5 days prior to the start of the studies. The animals had free access to food and water throughout the study.

### Preparation of inocula for lung infection models.

All medium was from BBL, Franklin Lakes, NJ. Bacteria were grown in 5% CO_2_ at 37°C overnight. *S. aureus* was cultivated on Trypticase soy agar (TSA), *S. pneumoniae* strains were grown on TSA with 5% sheep blood, and *H. influenzae* was grown on chocolate agar. The inoculum was prepared by suspending a portion of the overnight growth in sterile saline solution to achieve an optical density of 0.1 at 625 nm. The culture was subsequently diluted in brain heart infusion (BHI) medium to achieve each inoculum. For preparation of* H. influenzae* for intratracheal infections, 1% molten BHI agarose was added to the inocula and the 0.5% molten agarose bacterial suspension was maintained in a 42°C water bath throughout the infection procedure. All inoculation was done under conditions of light anesthesia (4.5% isoflurane with 1.5 liters/min O_2_).

### Quantification of bacterial load in lung.

At the time of bacterial burden determination, animals were euthanized by CO_2_ inhalation, the lungs were aseptically removed, weighed, and homogenized in sterile saline solution, and the homogenate was serially diluted and plated on TSA (MRSA and *S. pneumoniae*) or chocolate agar (*H. influenzae*). CFU per gram of lung were calculated after overnight incubation of plates at 37°C in 5% CO_2_.

### Neutropenic mouse lung infection model.

BALB/c mice (18 to 20 g, *n* = 5 to 6 per group) were rendered neutropenic through two consecutive intraperitoneal injections of cyclophosphamide of 150 and 100 mg/kg of body weight on days −4 and −1, respectively, and were inoculated intranasally on day 0 with 50 μl of bacterial inoculum. Mice received drug formulated in sterile 0.9% saline solution at 10 ml/kg via tail vein i.v. injection or via oral gavage in water at 2 and 12 h postinfection, except for linezolid, which was given only by oral gavage at 2 and 12 h postinfection. Bacterial burden in lung was determined at pretreatment and 24 h after initiation of treatment. The MIC values for TP-271 and linezolid against tetracycline-resistant *tet*(M) MRSA strain SA191 were 0.25 and 2 µg/ml, respectively. The MIC values for TP-271 and linezolid against tetracycline-resistant *tet*(M) *S. pneumoniae* strain SP160 were ≤0.016 and 1 µg/ml, respectively. Inocula were as follows (in CFU per mouse): for *S. aureus* SA191, 7.5 × 10^7^; and for *S. pneumoniae* SP160, 7.0 × 10^6^.

### Immunocompetent *S. pneumoniae* mouse lung infection model.

CD-1 mice (18 to 20 g, *n* = 5 to 6 per group) were inoculated intranasally with 1.6 × 10^7^ CFU of respiratory isolate *S. pneumoniae* SP514 at 50 μl per mouse. The MIC values for TP-271, linezolid, and clarithromycin were ≤0.008, 0.5, and ≤0.008 µg/ml, respectively. Mice were dosed p.o. with 30 mg/kg TP-271 or linezolid or with 5 mg/kg clarithromycin in a volume of 10 ml/kg at 5, 24, and 36 h postinfection. Bacterial burden in lung was determined at pretreatment and 48 h following initiation of treatment.

### Immunocompetent *H. influenzae* rat lung infection model.

Male Sprague-Dawley rats (175 to 200 g, *n* = 5 to 8 per group) were infected intratracheally with 6.5 × 10^7^ CFU per rat of ampicillin-resistant respiratory isolate *H. influenzae* HI551 at 0.5 ml. The MIC values for TP-271 and azithromycin were ≤0.016 and 0.5 µg/ml, respectively. At 5, 24, and 48 h, rats were dosed with a volume of 5 ml/kg containing TP-271 p.o. at 100 mg/kg or i.v. at 25 mg/kg; azithromycin was dosed p.o. at 50 mg/kg in a volume of 10 ml/kg. Bacterial burden in lung was determined at pretreatment and at 72 h following initiation of treatment.

### Pharmacodynamic analysis.

The mean log_10_ CFU count per gram of tissue obtained at the start of treatment group (T = 0 h) was subtracted from the log_10_ CFU count per gram of tissue obtained for each individual animal in the treatment groups to determine the change in log_10_ CFU per gram of tissue at the end of treatment. The mean change in log_10_ CFU per gram of tissue ± standard deviation was determined for the vehicle control, comparator(s), and TP-271 efficacy groups and graphed using GraphPad Prism version 4.03.
